# Shift of Feeding Mode in an Epizoic Stalked Barnacle Inducing Gall Formation of Host Sea Urchin

**DOI:** 10.1016/j.isci.2020.100885

**Published:** 2020-02-26

**Authors:** Luna Yamamori, Makoto Kato

**Affiliations:** 1 Graduate School of Human and Environmental Studies, Kyoto University, Sakyo, Kyoto 606-8313, Japan

**Keywords:** Biological Sciences, Zoology, Genetics, Phylogenetics

## Abstract

Among diverse stalked barnacles, *Rugilepas pearsei* (Thoracica: Cirripedia: Arthropoda) is a rare unique species that is associated with echinoids and has highly atrophied cirri. We rediscovered the barnacle for the first time from description and verified that the barnacles live obligately in half-open galls formed on the test of the sea urchin *Echinothrix diadema* (Diadematidae: Echinodermata). A molecular phylogenetic analysis demonstrated that the obligate association with echinoids derived from epizoic life on crustaceans. A stable isotope analysis suggests that the barnacle feeds on particulate organic matter (POM) without parasitizing the host echinoids. These findings suggest that the host shift caused losses of plates and feather-like cirri, changes in the attachment device from cementation to anchoring, and a shift in feeding mode from filter feeding to POM collection. The barnacle's epizoic, superficially sub-endozoic, communal life in stout but narrow galls causes repetitive reproduction at the cost of reduced growth.

## Highlights

• *Rugilepas* is the first stalked barnacle that induces gall formation on echinoids
• The epizoic barnacle feeds on particulate organic matter by using atrophied cirri
• The obligate association with echinoids derived from epizoic life on crustaceans
• The host shift caused changes of morphology, attaching device, and feeding mode

## Graphical Abstract

**Figure:**
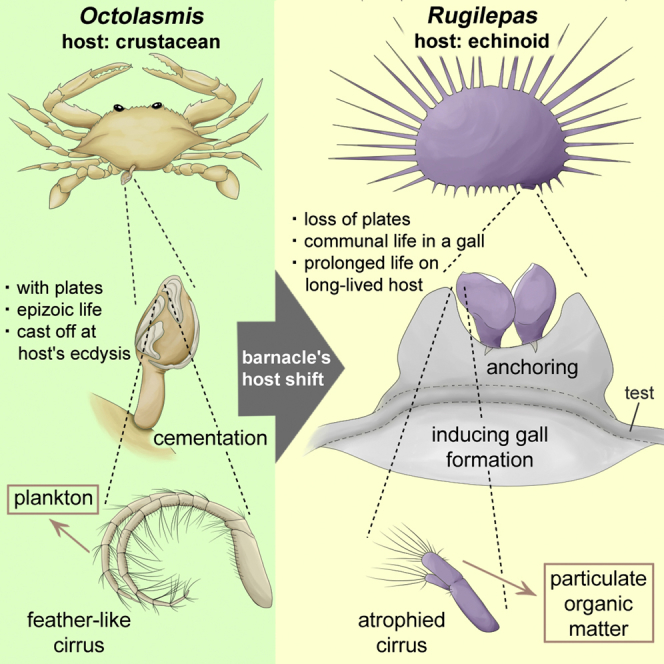

## Introduction

Most stalked barnacles (Thoracica: Cirripedia) are sessile suspension feeders that live attached to hard substrata or the exoskeletons/epithelia of diverse marine animals (Darwin, 1851), but some of these cirripedes parasitize their host animals such as annelids (Day, 1939) and sharks (Johnstone and Frost, 1927, Rees et al., 2014) by embedding a root-like organ into the host body. Other epizoic barnacles living on jellyfish (Pagès, 2000) and sea anemones (Yusa et al., 2001) have different parasitic feeding modes that are facilitated by shifting their attachment and feeding devices.

*Rugilepas pearsei* (Microlepadidae: Lepadiformes) is a rare naked stalked barnacle with atrophied cirri, and it lives in a symbiotic relationship with echinoids (Grygier, 1991), but its feeding habit is poorly understood. Because *R. pearsei* has not been recorded since its original description, rediscovery of the barnacle has been kept waiting to answer the questions: how the association with echinoids derived, which prey/substance the barnacle feeds on, and how shift of feeding mode has occurred in a lineage of epizoic suspension feeders. By conducting extensive search for the barnacle, morphological and ecological observations, a molecular phylogenetic analysis, and a stable isotope analysis, we explored the evolutionary trajectory of the echinoid-symbiotic barnacle.

## Results and Discussion

### Epizoic Life in Galls on Echinoids

We rediscovered *R. pearsei* on a coral reef off Okinawa Island, Japan, and verified that it is an obligate semi-endozoic animal living in half-open galls formed on the sea urchin *Echinothrix diadema* (Diadematidae: Echinodermata), which has venom gland at the point of the secondary aboral spine tip (von Reumont et al., 2014). This is the first report of a stalked barnacle inducing gall formation on echinoids.

**Figure 1:**
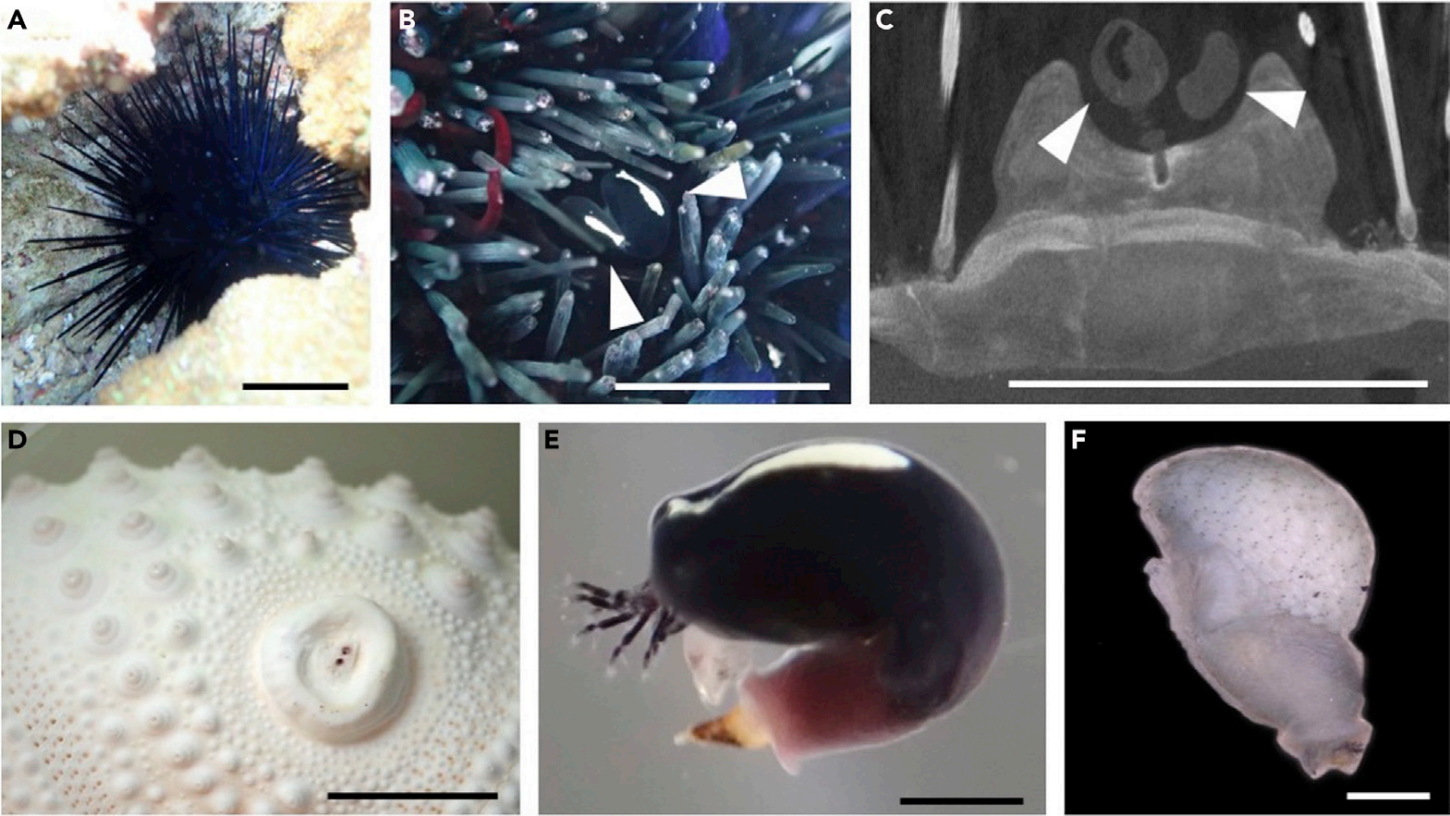
Habit, Morphology, and Phylogeny of *Rugilepas pearsei* (A) A living individual of *Echinothrix diadema* inhabiting crevice of coral reef. (B) Two living barnacles (arrowheads) attached to the base of a semi-open gall formed on the test of the host sea urchin *Echinothrix diadema*. (C) Computed tomography (CT) image of a gall inhabited by two barnacle individuals (arrowheads). (D) Undersurface of a bleached test of a galled sea urchin. (E) Lateral view of a detached individual with extended cirri. (F) Lateral view of a brooding individual after carapace removal. Scale bars: 10 cm in (A), 1.0 cm in (B)–(D), and 1.0 mm in (E) and (F). See also Figure S1 and Table S1.

We performed an extensive search for this barnacle on a coral reef off Manzamo and Bise on Okinawa Island and found it only in galls on *Echinothrix diadema* (Figures 1A and 1B). The rate of parasitism was 9.3% on *Et*. *diadema* (43 sea urchins examined) and 0% on *Et*. *calamaris* (56 sea urchins) and other *Echinometra* species (100 sea urchins each). All of the barnacles on *Et*. *diadema* were found living communally in semi-open galls formed in the interambulacral areas on the oral side of the sea urchin. We found two to four barnacles growing in clumps at the base of each gall. Computed tomography (CT) showed that the side and bottom walls of the gall tests were thickened markedly. The claw-like peduncular attachment organs of the barnacles were anchored deeply in the thickened basal area of the sea urchin test (Figure 1C). Compared with other areas of the test, the spines around the galls were highly modified, in that thick primary spines had been replaced with thin, poisonous secondary spines (Figure 1D). The barnacle color matched that of the host exactly. Galls were surrounded by the secondary spines of the host sea urchin, suggesting that the barnacles defend against predators by their protective coloration and by location of the stout galls on the lower sides of the sea urchin adpressed to the hard substratum. Life in such a protected location may have allowed the barnacle to lose its protective plates via evolution (Figure 1E).

All of the barnacles we found were hermaphroditic adults with short penises. All galls were inhabited by two to four barnacles but no dwarf male, suggesting that reciprocal insemination occurs within each gall. The fact that all the barnacles in a gall are similar in size (Table S1) suggests that a few cypris larvae have settled almost simultaneously. Eggs or stage I nauplius larvae were brooded inside the mantle sac cavities of all barnacles collected (Figure 1F). The larvae were tadpole-like with tapering tails and pairs of long frontolateral horns alongside the bodies. These observational data suggest that the barnacles copulate with each other within a single gall and continue to reproduce at the cost of reduced body growth within the confines of the gall. The prolonged communal life on long-lived host contrasts with short life of crustacean-epizoic barnacles, which are cast off along with the exuvia at hosts' ecdysis (Kobayashi and Kato, 2003).

### Feeding Habit of the Epizoic Barnacle in a Gall

**Figure 2:**
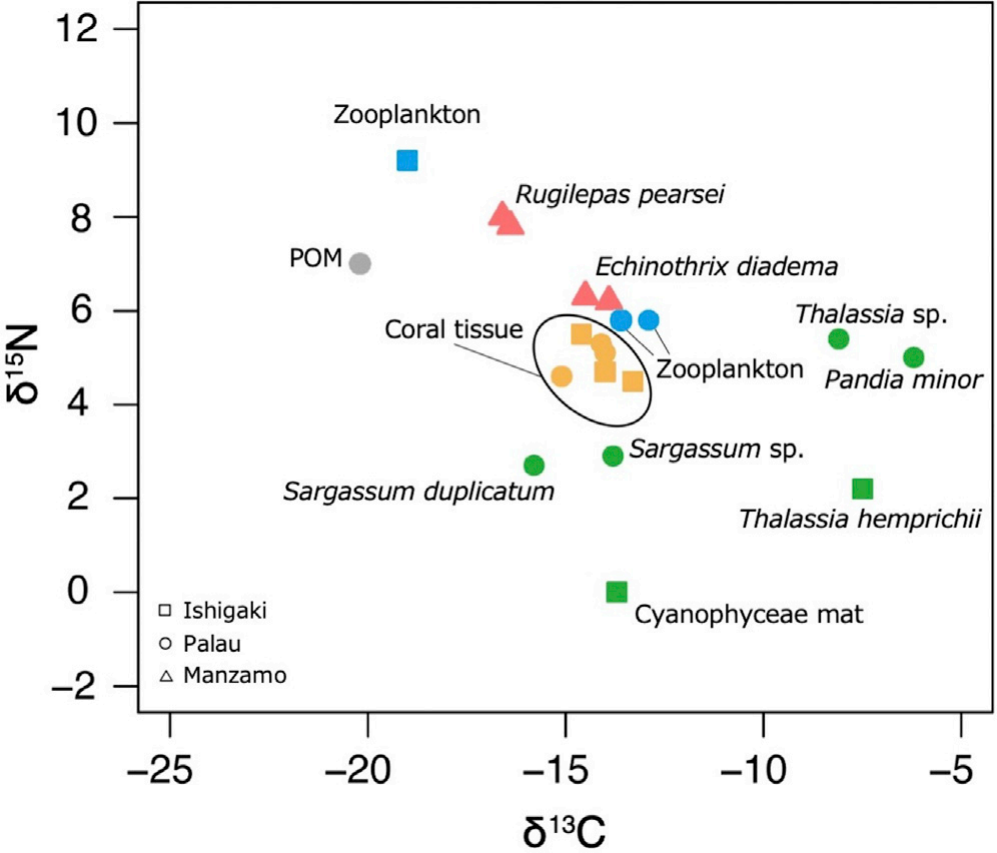
δ^13^C and δ^15^N Values of *Rugilepas pearsei* and Its Host Sea Urchin, *Echinotrhrix diadema*, Collected off Manzamo, Okinawa Island (This Study), and of Diverse Organisms and Organic Materials Collected in the Ishigaki and Palau Coral Reef Ecosystems. Producers are colored green, zooplankton blue, corals orange, benthic consumers red, and particulate organic matter (POM) gray. Superimposition of the data suggests that *Echinothrix diadema* feeds on corals and *Rugilepas pearsei* on POM.

Although the cirri were greatly atrophied, they were actively beating. During beating, the cirri protruded only slightly from the capitulum (Figure 1E); the strokes of individual cirri were not rhythmical. A stable isotope analysis of the ^13^C/^14^C and ^14^N/^15^N ratios of the barnacles and host sea urchins showed that they belonged to different food chains. Superimposition of the data onto the community-level datasets collected from coral reef ecosystems off Ishigaki Island and Palau (Yamamuro et al., 1995) suggested that the sea urchin feeds on corals and that the barnacle feeds on particulate organic matter (POM), partially decomposed detritus in water, without parasitizing the host echinoids (Figure 2).

### Evolutionary Shift of Feeding Mode

**Figure 3:**
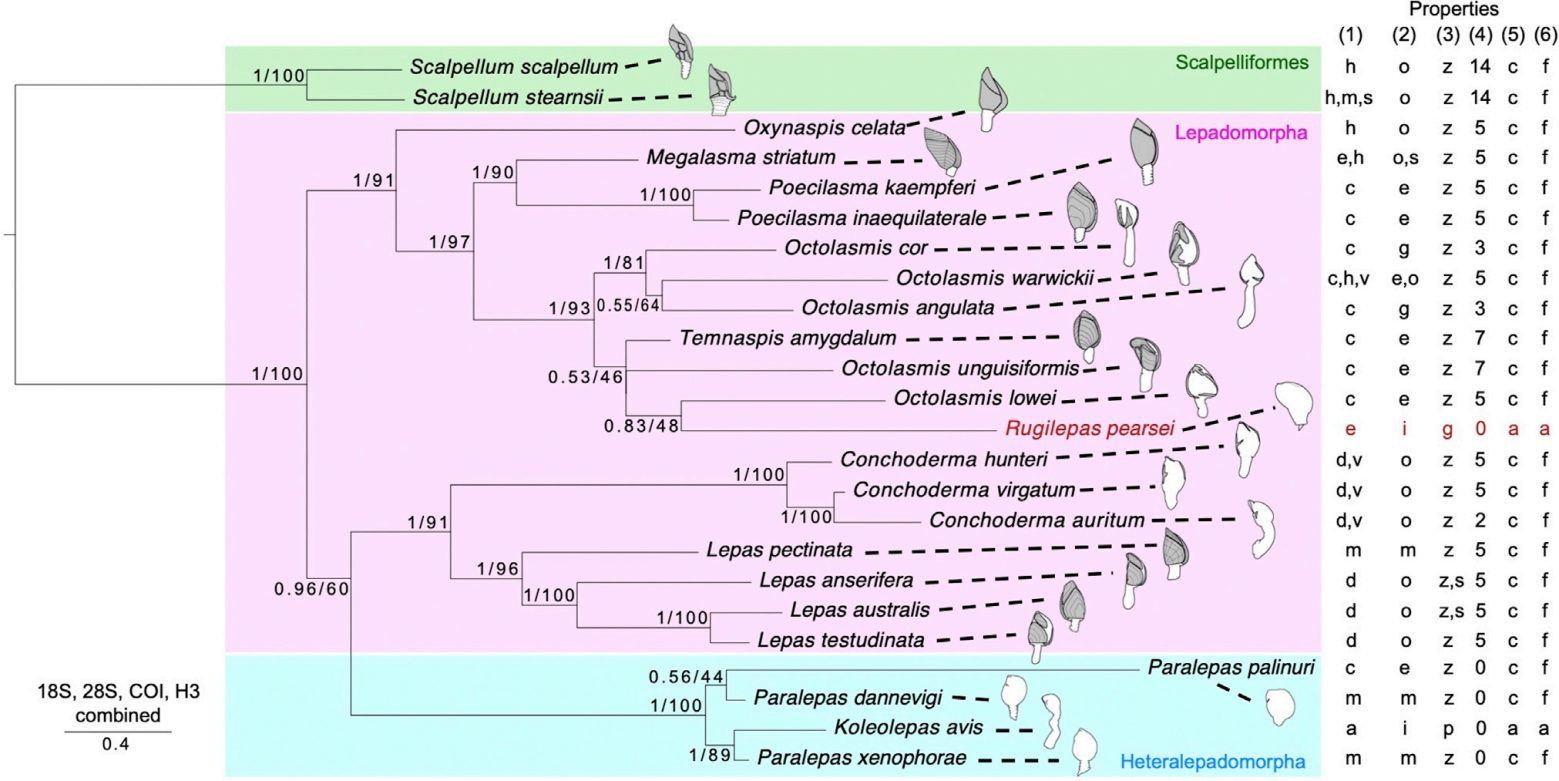
Bayesian Tree of Lepadomorpha and Heteralepadomorpha Species Based on the 18S rRNA, 28S rRNA, CO1, and H3 Gene Sequences The numbers at the nodes are (1) Bayesian posterior probability values and (2) maximum likelihood bootstrap support values. Illustration of the ecological habitus (plates are shaded) and the following six morphological/ecological properties of the barnacle species shown adjacent to the species binomials: (1) the dominant attachment host/substratum (a, Anthozoa; c, crustacean; d, driftage matter; e, echinoid; s, abiotic hard substratum; m, Mollusca; h, Hydrozoa; v, vertebrate); (2) attachment site (e, exoskeleton; g, gill; i, inside of host body; m, molluscan shell; o, outside surface; s, spine); (3) habit (g, sub-endozoic in a gall; p, parasitic; s, sessile on abiotic substratum, z, epizoic); (4) number of plates; (5) attachment device (a, anchoring; c, cementing); and (6) morphology of cirri. (a, atrophied; f, feather-like). See also Table S2.

Using the 18S rRNA, 28S rRNA, CO1, and histone 3 (H3) sequences, we performed molecular phylogenetic analyses of *R*. *pearsei* and related taxa belonging to Heteralepadomorpha and Lepadomorpha (Table S2). An analysis based on all markers combined (18S + 28S + CO1 + H3) (Figure 3) suggests that (1) *R*. *pearsei* belongs to Poecilasmatidae (Lepadomorpha) and (2) the genus *Rugilepas* has diverged from a group of *Octolasmis* species, most of which are epizoic on crustaceans. Since *Rugilepas* was originally described as Microlepadidae in Heteralepadomorpha, our result also suggested that Heteralepadomorpha is polyphyletic, as suggested by Herrera et al. (2015) and Perez-Losada et al. (2008). The character distribution in the phylogenetic tree suggests that the evolutionary transition involved the following: loss of shell plates, changes in the attachment device from cementation to anchoring, a shift in the feeding mode from filter feeding to POM collection, and a shift in the feeding device from feather-like cirri to atrophied stout cirri. Reduction of shells have occurred several times in epizoic barnacles in Poecilasmatidae, suggesting that epizoic habit eliminated the need for armored body, especially in *Rugilepas* living in armored gall. Although the shift from cementation to anchoring has occurred in coral-epizoic barnacles and shark-parasitic barnacles, cement gland is still functional at least in juvenile stages (Brickner and Høeg, 2010, Rees et al., 2014). Reduction of cirri may have been driven over evolution by the extremely cramped habitat in a narrow adpressed gall, in which filter feeding would be severely hampered. The shortened cirri would function to collect POM and to ventilate mantle cavity to the benefit of the brooded embryos and larvae. In this phylogenetic tree, degradation of cirri appears to have occurred twice: in the POM-feeding *Rugilepas* and in *Koleolepas*, a parasite of sea anemones, suggesting evolutionary flexibility of the filter-feeding mode in Pedunculata.

Although galls on echinoids are rare, several types of galls are formed by myzostomids, gastropods, and copepods (Jangoux, 1987), all of which are internal parasites. The gall formed by *Rugilepas* is unique because (1) it is induced by an epizoic cirripede and (2) the gall inducer is not a parasite of the host. The mechanism by which the barnacle cypris larvae on the sea urchin induce gall formation on the host echinoid remains unclear because echinoderms generally have complex, sophisticated immune systems (Smith et al., 2010).

### Limitation of the Study

Because of the difficulties of collecting samples, the phylogenetic tree is constructed from a single specimen.

## Methods

All methods can be found in the accompanying Transparent Methods supplemental file.

## Data and Code Availability

The DNA sequences gained in this study were deposited to GenBank, and the accession numbers are listed in Table S1.

## Supplemental Information

Document S1. Transparent Methods, Figure S1, and Tables S1 and S2
