# Supplementary material for: Shift of Feeding Mode in an Epizoic Stalked Barnacle Inducing Gall Formation of Host Sea Urchin
Source: iScience. 2020 Feb 26;23(3):100885. doi: 10.1016/j.isci.2020.100885 (PMC7113561; doi:10.1016/j.isci.2020.100885)
Supplement: Document S1. Transparent Methods, Figure S1, and Tables S1 and S2 [file mmc1.pdf]

iScience, Volume *23*

Supplemental Information

Shift of Feeding Mode in an Epizoic Stalked Barnacle Inducing Gall Formation of Host Sea Urchin

Luna Yamamori and Makoto Kato

Supplemental Figures

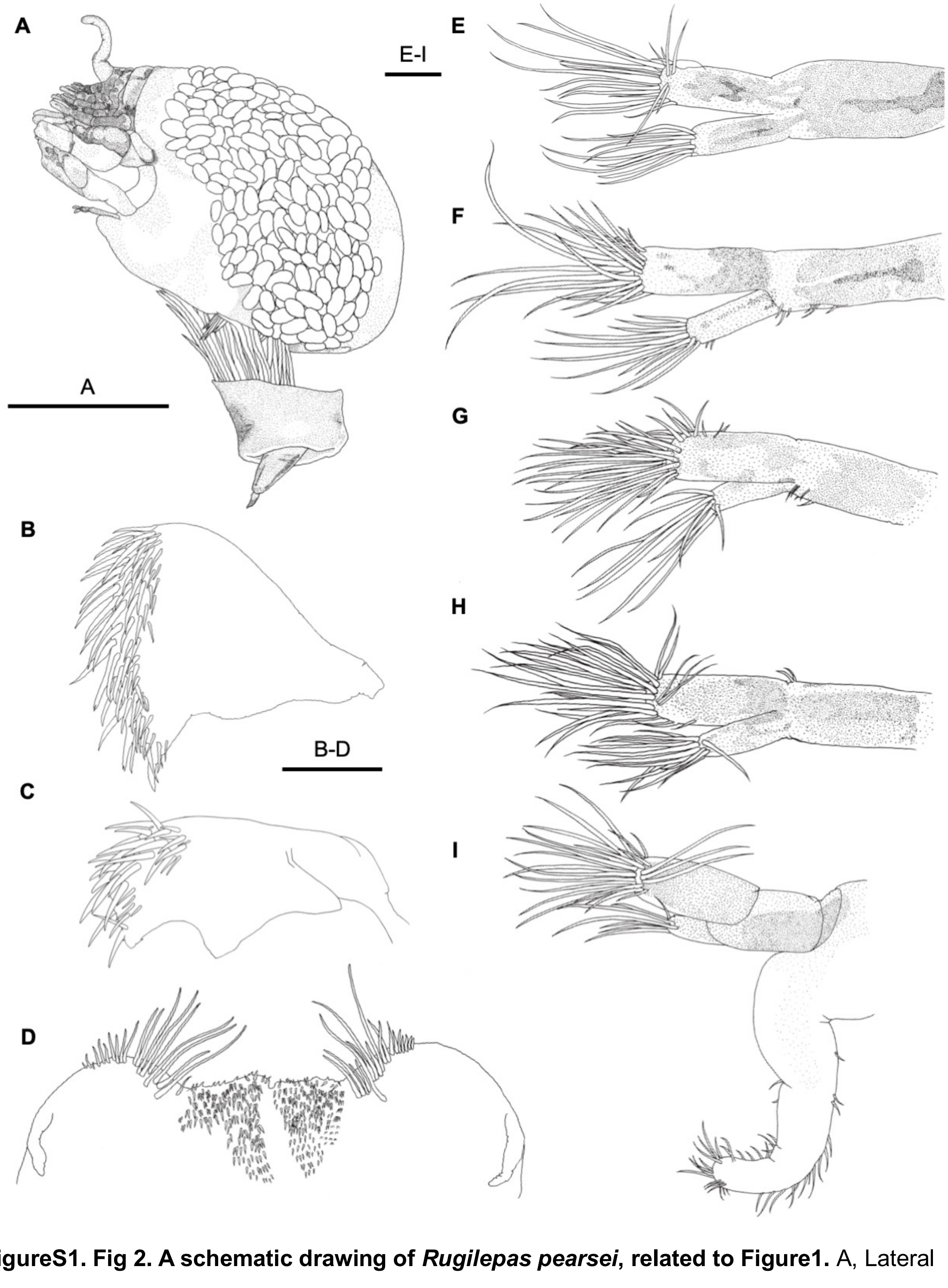

FigureS1. Fig 2. A schematic drawing of *Rugilepas pearsei*, related to Figure1. A, Lateral view of the barnacle body with its carapace removed; B, mandible; C, maxillule; D, labrum; E, cirrus I; F, cirrus II; G, cirrus III; H, cirrus IV; I, cirrus V. Scale bars: A, 1 mm; B–I, 0.1 mm.

Table S1. Measurement of each gall and barnacles inside, related to Figure1.

Gall Number	Sea urchin diameter (cm)	Gall diameter (mm)	Number of barnacles in a gall	Capitulum length of barnacle (mm)			
1	9.37	4.5	4	3.41	3.33	3.35	3.44
2	10.9	4.3	2	3.52	3.47		
3	11.1	4.1	3	3.43	3.43	3.31	
4	10.1	4.2	3	3.34	3.54	3.33	

Table S2. A list of lepadomorph species analyzed for genetic sequence of four genes (28S rRNA, 18S rRNA, COI and H3), related to Figure3.

Suborder	Family	Species	Specimen	Genbank #				Locality	References
			Catalogue #	28S rRNA	18S rRNA	COI	H3		
Scalpelliformes	Scalpellidae	*Scalpellum scalpellum*	Genbank	EU082307	EU082388	KT209468	EU082347		Høeg et al., 2016
		Scalpellum stearnsii	Genbank	NA	KF484143	KF484216	KF484288		Chan, 2009
Lepadomorpha	Lepadidae	*Lepas anserifera*	Genbank	EU082404	GU993686	EU082363	KY944875		Whitehead et al., 2010
		Lepas australis	Genbank	EU082324	FJ906777	GU993642	EU082364		Hinojosa et al., 2006
		Lepas pectinata	Genbank	EU082322	FJ906774	MK308245	EU082362		Hinojosa et al., 2006
		Lepas testudinata	Genbank	EU082325	EU082406	KC138478	EU082365		Whitehead et al., 2010
		Conchoderma auritum	Genbank	EU082320	EU082401	KU204353	EU082360		Christensen, 1985
		Conchoderma hunteri	Genbank	NA	KF484140	KF484213	KF484285		Yamato et al., 1996
		Conchoderma virgatum	Genbank	EU082402	NA	KC138464	EU082361		Yamato et al., 1996
	Oxynaspididae	*Oxynaspis celata*	Genbank	EU082412	NA	KF484231	EU082371		Jones et al., 2000
	Poecilasmatidae	*Megalasma striatum*	Genbank	EU082411	NA	KF484231	EU082371		Jones et al., 2000
		Octolasmis angulata	Genbank	AB551727	NA	KC138498	NA		Jones et al., 2000
		Octolasmis cor	Genbank	EU082407	NA	MH753552	EU082366		Jones et al., 2000
		Octolasmis lowei	Genbank	L26518	NA	NA	NA		Jones et al., 2000
		Octolasmis unguisiformis	YN190121	LC467957	LC467955	LC467960	LC467958	(1)	Kobayashi and Kato, 2003
		Octolasmis warwickii	Genbank	AB551728	NA	NA	NA		Jones et al., 2000
		Poecilasma kaempferi	Genbank	EU082410	NA	NA	EU082369		Jones et al., 2000
		Poecilasma inaequilaterale	Genbank	AY520654	AY520754	NA	AY520722		Williams and Brown, 1972
		Temnaspis amygdalum	Genbank	AB551730	NA	NA	NA		Jones et al., 2000
		Rugilepas pearsei	KUZ 2473	LC467956	LC467954	LC467959	NA	(2)	Grygier, 1991
Heteralepadomorpha	Koleolepadidae	*Koleolepas avis*	Genbank	NA	AB551734	NA	NA		Yusa et al., 2001
	Heteralepadidae	*Paralepas dannevigi*	Genbank	EU082318	EU082399	NA	EU082358		Brock, 1922
		Paralepas palinuri	Genbank	NA	AF057561	NA	NA		Newman, 1960
		Paralepas xenophorae	Genbank	NA	AB551733	NA	NA		Newman, 1960

(1), Yanyu, Kagoshima, Japan; (2), Manzamo, Okinawa, Japan.

Transparent Methods

Study site

Our study was conducted on a coral reef off Manzamo in Okinawa Prefecture, Japan. No specific permissions were required for sampling at this location, and neither endangered nor protected species were involved in the field study. Most corals in the inner lagoon were dead. Four *Echinometra* species (*Em. mathaei, Em. oblonga, Em. ryukyuensis*, and *Em. tsumajiro*) burrowed trenches on the dead corals. The black sea urchin *Stomopneustes variolaris* inhabited the undersurfaces of the corals. Most of the corals in the outer lagoon were alive. *Echinothrix diadema* occurred on the undersurfaces of living corals. On the wave-swept reef edge, the slate pencil urchin *Heterocentrotus mammillatus* inhabited narrow crevices in the reefs. In July 2017, we found the unusual gall-inducing stalked barnacle *Rugilepas pearsei* on the test of *Et. diadema*. In May 2018, we conducted an extensive search for this barnacle on the dominant sea urchin species *Em. tsumajiro*, *S. variolaris*, and *Et. diadema.* We collected 100 individuals of *Em. tsumajiro* and *S. variolaris* and 43 individuals of *Et. diadema.* Additionally, we also searched for the stalked barnacle on 56 individuals of *Et. calamaris* found on a coral reef off Bise, which is located northward from Manzamo in Okinawa Prefecture. All sampled sea urchins were examined for symbionts; only those harboring stalked barnacles were collected and preserved in 99% and 70% ethanol for molecular phylogenetic analysis and observations of morphology and gastric contents, respectively.

Morphological observations

One individual of *Et. diadema*, which bore a gall harboring two stalked barnacles, was preserved directly in 70% ethanol and examined by computed topography at the Japan Multi-Industrial Company Cooperation, Kanagawa. After computed topography scanning, the

stalked barnacles were detached from the host sea urchin. To examine the surface morphology of the gall, the sea urchin was soaked in an undiluted sodium hypochlorite solution for ~2 h until all of the spines and tube feet had detached from the test.

Trophic analysis

To explore the trophic interaction between the barnacle and host sea urchin, four barnacles that had been fixed immediately after collection were dissected, and their gastric contents were observed under an optical microscope. To determine their trophic levels in the food chain, we measured the stable isotope ratios of the barnacle and host. The muscle tissues (30 mg dry weight) of two barnacle individuals and the tube feet of two *Et. diadema* individuals were subjected to stable isotope analysis at the Isotope Research Institute, Tokyo to determine the $^{13}C/^{14}C$ and $^{14}N/^{15}N$ ratios.

Molecular phylogenetic analysis

We performed direct PCR using the Ampdirect® Plus reagent (SHIMADZU) to amplify the DNA from the ethanol-preserved skin (3–5 mg dry weight) of two stalked barnacle species: *Octolasmis unguisiformis* and *R. pearsei*. The tissue was dried, soaked in 50 μl lysis buffer (20 mM Tris-HCl at pH 8.0, 5 mM EDTA, 400 mM NaCl, 3% SDS, and 200 μmol/ml proteinase) and incubated at 60°C for 12 h. PCR was used to amplify four genes: 18S rRNA (~1850 bp amplicon) and 28S rRNA (1600 bp amplicon) using previously described primers (Rees et al., 2014), histone 3 (H3; 350 bp amplicon) using the universal primers H3F/H3R (Colgan et al., 1998), and mitochondrial CO1 (700 bp amplicon) using the universal primers LCO1490/HCO2198 (Vrijenhoek, 1994). The purified PCR products (≥50 ng/μl) were subjected to direct sequencing (Macrogen, Kyoto, Japan) using the primer sets described above. The sequences obtained were

deposited in the DDBJ/EMBL/GenBank databases under the accession numbers listed in Table S1. The sequences obtained in this analysis and additional sequences provided by GenBank were aligned using the Muscle package (Edgar, 2004) implemented in Seaview software (Galtier et al., 1996; Gouy et al., 2010) with the default settings. We employed Gblocks v. 0.91b software (Castresana, 2000; Talavera and Castresana, 2007) to eliminate any ambiguously aligned regions of the 18S and 28S sequences. The sequence lengths of the 18S gene before and after Gblocks treatment were 1792 and 1753 bp, respectively, and the respective lengths of the 28S gene were 1720 and 1561 bp. The alignments of the CO1 and H3 genes did not contain any insertions or gaps and were therefore unambiguous. Bayesian and maximum likelihood phylogenetic analyses were performed on the combined data set (18S + 28S + CO1 + H3) using the MrBayes v. 3.1.2 (Ronquist and Huelsenbeck, 2003) and RAxML v. 7.4.2 (Stamatakis, 2006) packages implemented in raxmlGUI v. 1.31 software (Silvestro and Michalak, 2012). We selected the model GTRGAMMA for the RAxML analysis and used Kakusan4 software (Tanabe, 2011) to select the appropriate models for the MrBayes analysis. The models selected for MrBayes analysis were GTR_GAMMA for the 18S and 28S genes, HYK85_GAMMA, GTR_GAMMA and F81_GAMMA for each codon of the CO1 gene, GTR_GAMMA for the first codon of the H3 gene, and J69_Homogenoeous for the second and third codons of the H3 gene. Two independent runs of the Metropolis-coupled Markov chain Monte Carlo procedure were performed simultaneously for 5,000,000 generations. Voucher specimens of the stalked barnacles examined for taxonomic assignment were deposited in the Kyoto University Museum.

Data and Software Availability

The gene sequences gained in this study are listed in the Table S2.

Supplemental References

Colgan, D.J., McLauchlan, A., Wilson, G.D.F., Livingston, S.P., Edgecombe, G.D., Macaranas, J., Cassis, G., and Gray, M.R. (1998). Histone H3 and U2 snRNA DNA sequences and arthropod molecular evolution. Australian Journal of Zoology 46, 419–437.

Vrijenhoek, R. (1994). DNA primers for amplification of mitochondrial cytochrome c oxidase subunit I from diverse metazoan invertebrates. Mol Mar Biol Biotechnol 3, 294–299.

Edgar, R.C. (2004). MUSCLE: a multiple sequence alignment method with reduced time and space complexity. Bmc Bioinformatics 5, 1–19.

Galtier, N., Gouy, M., and Gautier, C. (1996). SEAVIEW and PHYLO_WIN: Two graphic tools for sequence alignment and molecular phylogeny. Computer Applications in the Biosciences 12, 543–548.

Gouy, M., Guindon, S., and Gascuel, O. (2010). SeaView Version 4: A Multiplatform Graphical User Interface for Sequence Alignment and Phylogenetic Tree Building. Molecular Biology and Evolution 27, 221–224.

Castresana, J. (2000). Selection of conserved blocks from multiple alignments for their use in phylogenetic analysis. Molecular Biology and Evolution 17, 540–552

Talavera, G., and Castresana, J. (2007). Improvement of phylogenies after removing divergent and ambiguously aligned blocks from protein sequence alignments. Systematic Biology 56, 564–577.

Ronquist, F., and Huelsenbeck, J.P. (2003). MrBayes 3: Bayesian phylogenetic inference under mixed models. Bioinformatics 19, 1572–1574.

Stamatakis, A. (2006). RAxML-VI-HPC: Maximum likelihood-based phylogenetic analyses with thousands of taxa and mixed models. Bioinformatics 22, 2688–2690.

Silvestro, D., and Michalak, I. (2012). raxmlGUI: a graphical front-end for RAxML. Organisms Diversity & Evolution 12, 335–337.

Tanabe, A.S. (2011). Kakusan4 and Aminosan: two programs for comparing nonpartitioned, proportional and separate models for combined molecular phylogenetic analyses of multilocus sequence data. Molecular Ecology Resources 11, 914–921.

Høeg, J.T., Yusa, Y., and Dreyer, N. (2016). Sex determination in the androdioecious barnacle *Scalpellum scalpellum* (Crustacea: Cirripedia). Biological Journal of the Linnean Society 118, 359–368.

Chan, B.K.K. (2009). Shallow water and deep-sea barnacles (Crustacea: Cirripedia: Thoracica) collected during the philippine panglao 2005 expedition, with descriptions of two new species. Raffles Bulletin of Zoology, 47–82.

Whitehead, T.O., Biccard, A., and Griffiths, C.L. (2011). South african pelagic goose barnacles (Cirripedia, Thoracica): substratum preferences and influence of plastic debris on

abundance and distribution. Crustaceana 84, 635–649.

Hinojosa, I., Boltana, S., Lancellotti, D., Macaya, E., Ugalde, P., Valdivia, N., Vasquez, N., Newman, W.A., and Thiel, M. (2006). Geographic distribution and description of four pelagic barnacles along the south east Pacific coast of Chile – a zoogeographical approximation. Revista Chilena De Historia Natural 79, 13–27.

Christensen, I. (1985). First record of gooseneck barnacles (Conchoderma auritum) on a minke whale (*Balaenoptera acutorostrata*). International Council for the Exploration of the Sea C.M. 1985/N:9, 1–3.

Yamato, S., Yusa, Y., and Tanase, H. (1996). Distribution of two species of *Conchoderm*a (Cirripedia: Thoracica) over the body of a sea snake, *Laticauda semifasciata* (Reinwardt), from the Kii Peninsula, Southwestern Japan. Publications of the Seto Marine Biological Laboratory 37, 337–343.

Jones, D.S., Hewitt, M.A., and Sampey, A. (2000). A checklist of the Cirripedia of the South China Sea. Raffles Bulletin of Zoology, 233–307.

Kobayashi, C., and Kato, M. (2003). Sex-biased ectosymblosis of a unique cirripede, *Octolamis unguilsiformis* sp. nov., that resembles the chelipeds of its host crab, *Macrophthalmus milloti*. Journal of the Marine Biological Association of the United Kingdom 83, 925–930.

Williams, A.B., and Brown, W.S. (1972). Notes on Structure and Parasitism of *Munida ris* A. Milne Edwards (Decapoda, Galatheidae) from North Carolina, USA. Crustaceana, 303–308.20.

Brock, H. (1922). Studies on Pacific cirripeds. Papers from Dr. Th. Mortensen's Pacific Expedition 1914–1916, No. X. Vedensk. Meddel. Dansk Naturhist., Foren. Kobenhavn 73, 215–358.

Newman, W.A. (1960). Five pedunculate cirripeds from the western Pacific, including two new forms. Crustaceana, 100–116.
